# Job stress and emotional exhaustion at work in Spanish workers: Does unhealthy work affect the decision to drive?

**DOI:** 10.1371/journal.pone.0227328

**Published:** 2020-01-13

**Authors:** Francisco Alonso, Cristina Esteban, Adela Gonzalez-Marin, Elisa Alfaro, Sergio A. Useche

**Affiliations:** 1 DATS (Development and Advising in Traffic Safety) Research Group, INTRAS (University Research Institute on Traffic and Road Safety), University of València, Valencia, Spain; 2 University Center of Defense (Spanish Air Force Academy), Santiago de la Ribera, Murcia, Spain; 3 PRECOVIR (Risk Prevention in Road Behavior) Research Group, INTRAS (University Research Institute on Traffic and Road Safety), University of València, Valencia, Spain; Tongii University, CHINA

## Abstract

**Objectives:**

The purpose of this study was to assess the relationships among the following elements: unhealthy work indicators (job stress and emotional exhaustion at work), the decision to drive (or not), and driving crashes suffered by Spanish workers.

**Methods:**

For this cross-sectional study, a full sample of 1,200 Spanish drivers (44% women and 56% men) was used, their mean age being 42.8 years. They answered a questionnaire divided into three sections: demographic and driving-related data; burnout, job stress, and life stress; and self-reported road behaviors and driving safety indicators.

**Results:**

Overall, 41.6% of drivers reported emotional exhaustion at work. Furthermore, 80.2% of the participants showing substantial signs of job stress or exhaustion had experienced one or more important stressful life events during the previous year. Job stress was associated with the number of driving crashes suffered along the last 3 years. Also, and especially in situations where drivers admit not feeling well enough to drive, job stress and emotional exhaustion seem to be independent from the decision to drive, and from perceiving these variables as potential impairers of driving performance.

**Conclusions:**

First of all, this study showed a high prevalence of job stress and emotional exhaustion symptoms experienced at work by Spanish workers. Moreover, significant relationships were found among self-rated driving performance, workplace stress and burnout indicators, which suggests that job stress and emotional exhaustion levels may, indeed, impair driving performance, but they do not influence the decision to drive or not. In other words, even when they are significantly affected by job stress or emotional exhaustion at work, most Spanish drivers still drive.

## Introduction

Job stress (JS) has been pointed out by different studies as a relevant predictor of workers’ discomfort, morbidity and even mortality [1,2], and it is closely related to emotional exhaustion at work (EEW), one of the three key structural factors of the most accepted approach to burnout [3], which in turn increases the risk of accidents inside and outside the workplace [3,4]. On the other hand, there are many psychological and medical conditions that may substantially impair the driving performance. Nevertheless, it is relatively common to see how many drivers undervalue the impact of such factors on driving safety [5]. In this sense, the understanding of many health-related factors which affect the driving performance and the increase of the knowledge and awareness of drivers implies that relevant actions that can be taken, since they are tools which may strengthen road safety [6,7]. This is especially relevant when considering certain facts, such as that the prevalence of mental disorders is considerably frequent—and approximately a third of people will suffer from these disorders at some point of their lives- [8].

As for the occupational context, the National Institute for Occupational Safety and Health (NIOSH) considers that professional exhaustion can affect almost all the working population. Specifically, recent studies have revealed that a percentage between 25% and 30% of workers suffer from a high level of stress because of their jobs [9]. Furthermore, recent studies have established relationships between emotional exhaustion of workers and their driving behavior and performance. For instance, Li et al. [10] found that higher levels of EEW are positively and significantly linked to driving anger, and that EEW may be a relevant predictor of high-risk driving behaviors that may lead to traffic crashes; also, Montoro et al. [11] found significant associations between job stress and the rate of traffic fines received as a consequence of drivers’ road misbehaviors.

### The effort-reward imbalance (ERI) model as work-stress measurement tool, and its role in the study of workers’ health

A significant proportion of the research on job stress has been conducted following the *Effort-Reward Imbalance* (ERI) approach [12]. According to this model, workers do physical and psychological work, and, in exchange, they expect to obtain both intrinsic and extrinsic rewards (e.g., payment, job security, support, and respect) [13]. However, the imbalance in this mutual effort-reward ratio causes, as a result, the breach of the worker’s expectations together with other negative consequences, such as frustration, grief, and demoralization [14]; depression, or emotional fatigue [15,16]. In this sense, *reciprocity* represents the fundamental base of interpersonal behavior, in such a way that negative emotions appear when this behavior is damaged as a consequence of high efforts and lack of rewards. Smith, Roman, Dollard, Winefield & Siegrist [17] were the first ones to explore this aspect, and they found that ERI relates to the emotions experienced under a state of anger. Other previous works have also established relationships between work stress, as understood in the Job Demand-Control approach [18], and negative results in terms of health and road safety outcomes [19,20].

Research in many industrialized countries has shown a strong relation between job stress and health issues [21]. More recently, the research conducted by McLinton & Dollard [22] revealed that some negative effects derived from job stress can affect other activities, including driving. These studies found a close relationship between job stress and anger in the driving task, when these are associated with a highly excited state; the latter increases, in turn, the tendency to commit mistakes while detecting and processing information or making decisions both in the work environment and in the external facets of the daily life [23]. For instance, both Hoggan & Dollard [24] and McLinton & Dollard [22] found significant associations between work stress from the Effort-Reward Imbalance approach, being stress a factor potentially increasing road rage (measured as driving anger), and the subsequent driving behavior of -respectively- Australian and Japanese workers, through the statistical mediation of trait/general anger [22,24]. Consistently, job stress measured through other theoretical approaches different to the ERI model have also been related to risky road behaviors of drivers [11,25].

### Associations between emotional exhaustion at work, health and safety complaints of workers

Regarding the existing evidence in the working population, EEW has already been related to different adverse health consequences such as: depressive and anxious disorders [26,27], systematic headaches [28], a greater Odds Ratio (OR) for developing hypertension [29] gastro-intestinal diseases [30], musculoskeletal disorders [28], acute/chronic fatigue [31,32], numerous sleep problems [33–35], and a lower performance at work, in comparison to workforce groups that, on the other hand, have not reported burnout symptoms [36,37]. Empirically, some studies have found that burnout-related issues, such as EEW, may explain higher rates of job absenteeism and consistent turnover rates of workers [6,38,39], including the case of professional drivers, whose stressful work environment and role conflicts seem to be closely related to the appearance and prevalence of burnout-related symptomatology and other negative occupational and health outcomes [40,41].

Nevertheless, different systematic actions such as the assessment of tasks, a constant monitoring of workers’ mental health, the management of fatigue [26,42] and the increase and enhancement of peers’ social support, may strengthen the prevention and management of both job stress and burnout-related symptoms at the workplace [43,44]. The latter acquires special relevance when considering that, according to findings from other empirical studies, emotional exhaustion and its related psychosocial outcomes at work not only seem to affect the work-related performance, but -and as we have mentioned when addressing the findings of Li et al. [10]- to influence the workers’ outcomes in further spheres, such as decision making and safe behaviors at the wheel. In this regard, several studies have systematically shown how emotional exhaustion at work and job stress may affect the driving behavior, potentially influencing the occurrence of human-based traffic crashes linked to risky driving behaviors [45,46]. However, the evidence linking work environment settings, driving-related decision making and driving performance is still scarce [47,48], a fact that highlights the need of performing further empirical studies on this matter.

Thus, the core motivation to perform this study is the need of establishing relationships between two widely used indicators of unhealthy work (i.e., emotional exhaustion and job stress) and factors related to driving safety, such as the drivers’ decision to drive and their driving crashes, in order to strengthen the formulation and development of evidence-based interventions in road safety from the practice of occupational health.

### Objectives and hypotheses

The main purpose of this study was to assess the relationships among two unhealthy work indicators (job stress and emotional exhaustion at work, which is one of the core indicators of burnout), the decision to drive (or not), and the driving crashes suffered by Spanish workers. Considering the available evidence on the negative effects of these variables in driving safety, it is hypothesized that drivers experiencing higher levels of job stress and emotional exhaustion at work will be less aware of the risks of driving under adverse emotional states, thus tending to perform more risky road behaviors at the wheel and to suffer more crashes than drivers with lower levels of stress and exhaustion.

## Materials and methods

### Sample

The study sample was composed of a total of *n* = 1,200 Spanish drivers (56% men and 44% women) aged between 18 and 64 years, with a mean value of *M =* 42.8 (*SD* = 13.5) years. The sample size was calculated in order to keep an optimal proportionality with the quota of different population segments, according to variables such as age and sex. Demographic features and key driving habits and patterns of the sample are presented in Table 1.

**Table 1:** Demographic data and driving patterns of the sample.

Feature	Category	Frequency	Percentage
Sex	Female	534	44.5%
	Male	666	55.5%
Current work status	Active	835	69.6%
	Inactive (including sick leave, holidays, unemployed)	272	22.7%
	Householding	93	7.8%
Driving frequency	Daily	805	67.1%
	Almost daily	151	12.6%
	A few days a week	187	15.6%
	A few days a month	57	4.8%
Driving experience	< 1 year	44	3.7%
	1–2 years	80	6.7%
	3–10 years	277	23.1%
	11–20 years	300	25.0%
	21–30 years	284	23.7%
	> 30 years	215	17.9%
Main (most frequent) reason for driving	As part of my job	223	11.3%
	Commuting	167	16.4%
	Leisure/personal reason(s)	425	27.7%
	Indistinctly	376	44.6%
	Other	9	0.8%
Most frequent type of road	Urban road	422	35.2%
	Rural road	249	20.8%
	Highway‎/expressway	275	22.9%
	Various types	254	21.2%
Type of vehicle	Private car	1126	93.8%
	Motorcycle/moped/two-wheeled	54	0.8%
	Van	48	4.0%
	Heavy vehicle (truck, bus, freight)	16	1.4%

### Design, procedure and instruments

For this cross-sectional study, drivers were asked to complete a telephone-based survey, based on a representative national sample, and they were contacted through random digit dialing. The main participant-selection criteria were: first, drivers needed to be in possession of a valid license to drive motor vehicles; second, they needed to have been an active worker (at least) during the previous year; and third, they had to drive frequently. The surveys were carried out by means of Computer-Assisted Telephone Interviewing *(CATI)* system, fact that, in addition, allows researchers to solve potential doubts from participants during the data collection, minimizing data bias derived from the misunderstanding of questions or statements. Finally, the importance of providing honest answers to every question, and the inexistence of *right* or *wrong* answers, were remarked.

The data was collected, pooled and consolidated in the period 2008–2016, as part of the collaborative project “Attitudes” (corporate Social Responsibility program) of AUDI, in its section “*Road safety and health in Spain*”, that addressed the relationships between road safety and different health topics in Spain [49]. The global response rate was approximately 91.6%, considering that about 1,310 surveys were initially requested. Once the individuals agreed to partake in the study (please see *Ethics*), the survey was applied. This survey was structured in three sections, as follows:

In the first section, demographic data of the participants (age, sex, education, occupation) were collected. Also, participants were interviewed about their driving patterns (frequency and intensity, driving tenure or experience, type of vehicle usually driven).

As for the second section, it was composed of three self-report questionnaires for assessing, respectively, emotional exhaustion at work and job stress, and stressful life events. In the first case, the *Emotional Exhaustion* (EEW; 4 items; α = 0.867) sub-scale of the Maslach’s Burnout Inventory (MBI) [50] was used; it consists of a Likert scale (1 = highly disagree to 3 = highly agree) that presents different statements on emotional appraisals about the participant’s job (example item: *I feel emotionally drained by my job*). Two classification groups were established according to the sample distribution of the scores (based on the median or percentile 50 of the distribution): very low-mid symptoms of EEW—scores below 6, and with higher EEW symptoms—scores equal or higher than 6. For measuring job stress, a short version (10 items) of the Siegrist’s Effort-Reward Imbalance (ERI) Questionnaire [51] was used; it is a Likert scale (1 = highly disagree and 4 = highly agree) for assessing psychosocial risk factors at work, according to the stress under effort-reward imbalance model: extrinsic effort (3 items, α = 0.70; original α = 0.74) and reward (7 items; α = 0.80; original α = 0.79). This scale provides a standardized indicator of job stress (Effort-Reward Imbalance ratio) that can be correlated with other continuous variables. As a complementary indicator for assessing life-related stress, eight items from the Holmes & Rahes’ *Stressful Life Events Scale* [52] were included, examining different potential stressful situations (8 dichotomous items: Yes/No) that may occur in both daily life and the work environment (example item: *During the previous year*, *did you experience a major change in the health or behavior of a family member*?). It is important to point that, unlike the ERI, this scale is not meant to provide a stress index, but to summarize the adverse (potentially stressful) events that a person has suffered along the period of one year.

Finally, the third section contained a short inventory of deliberate risky driving behaviors (5 items; α = 0.68), for which the participants (all of them drivers) were asked to report how often (1 = never/almost never to 3 = very often) they perform five hazardous driving behaviors that may influence their driving safety outcomes: speeding, not keeping the safety distance, illegal overtaking, drinking and driving, and the use of cellphones while driving. In addition, participants were asked about their road safety outcomes (crashes suffered, and traffic fines received) during the last 3 years, and to state how often they did not feel in the proper condition to drive a motor vehicle. Also, they were asked about: a) the specific reason(s) and type of health disturbance(s), and b) the decision they made, in case they had to drive under the influence of the aforementioned discomfort. Finally, a supplementary set of items was given to participants, in order to determine: first, if they were aware and/or had received information about the potential risks of driving under adverse health conditions; and second, if they had decided to drive even when they were on sick leave.

### Ethics

The type of research described in this manuscript did not require the official intervention of the Ethics Committee in Experimental Research, as no personal data were used, and the participation was anonymous. However, the *Research Ethics Committee for Social Science in Health* of the University Research Institute on Traffic and Road Safety (INTRAS) of the University of València was consulted, certifying that the research subject to analysis responded to the general ethical principles currently relevant to research in Social Science, and the study received a favorable opinion to be carried out in Spain (IRB 000131117INT). Verbal consent was obtained from all participants before the carrying out the survey, but after informing respondents about the purposes of the study, its scientific character, the data protection laws and their anonymous involvement in the study. It is important to remark that surveys were recorded in order to document the partaker’s consent and the data retrieved, but no data allowing to identify participants (e.g., names, phone numbers, companies, addresses) were gathered, in order to ensure the anonymity of the survey.

### Statistical analysis (data processing)

First of all, we carried out the data curation, in order to enhance the basic aspects of the data that were to be analyzed. Once the data were clean and properly labelled, basic descriptive analyses were performed on the study sample in order to characterize the participants according to their demographic features and driving-related patterns. To do this, we used descriptive analyses, in order to obtain the basic scores for each one of the used scales, and frequency analyses for characterizing the prevalence of workplace stress and professional burnout-related issues. Pearson’s bivariate correlations were used to assess the association between the main study (quantitative) variables. Furthermore, Chi-square (*X*^*2*^) and cross-tabulation analyses were performed in order to identify significant trends in the association of categorical variables (i.e. characteristics and habits) of Spanish drivers composing the study sample. After the data collection, systematization and revision, statistical analyses were conducted by means of the © IBM SPSS (Statistical Package for Social Sciences) Software, version 24.0.

## Results

### Correlation analysis

The correlation analysis (see Table 2) allowed us to establish significant measures of association between study factors related to demographic features, driving habits, burnout and stress-related indicators, risky driving behaviors, and negative driving safety outcomes. As a result, interesting significant associations were found between pairs of variables.

**Table 2:** Bivariate correlations among main quantitative study variables (full sample).

		Mean	SD	2	3	4	5	6	7	8
**1**	Age (years)	42.90	12.51	.113**	-.057*	-.231**	.202**	-.181**	.068*	-0.004
**2**	Hours driving*day	4.20	1.23	-	0.048	0.047	-0.025	.059*	0.014	0.070*
**3**	Emotional Exhaustion at Work (EEW)	1.39	.46		-	.914**	-.145**	0.056	0.01	-0.018
**4**	Effort-Reward Imbalance ratio–job stress (JS)	1.14	.53			-	-.165**	.096**	0.016	-0.002
**5**	Stressful life events (SLE)	14.33	1.41				-	-.156**	-0.033	-0.043
**6**	Risky Driving Behaviors (RDB)	1.01	.91					-	.145**	.216**
**7**	Traffic crashes (3 years)	.70	.83						-	.115**
**8**	Traffic fines (3 years)	.29	.592							-

** Correlation is significant at 0.01 level (2-tailed)

* Correlation is significant at 0.05 level (2-tailed).

The age of drivers was significantly and negatively associated with both unhealthy work indicators (emotional exhaustion; *r* = -.057*, and job stress; *r* = -.231**), and positively associated to SLE (*r* = .202**). As for road behaviors, age negatively correlates with risky behaviors at the wheel (RDB); this means that older drivers tend to perform risky road behaviors less than younger ones.

Regarding variables related to the work environment (emotional exhaustion and job stress), it was found that EEW positively and significantly correlates to JS (*r* = .914**), and negatively to SLE (*r* = -.145**). Furthermore, JS also presents a significant and positive covariation with the self-reported risky driving behaviors (*r* = .096**). Finally, RDBs were positively associated with the driving crashes suffered by drivers (*r* = .145**) and traffic fines received while driving (*r* = .216**) during the last three (3) years.

### Demographic-based differences

**Fig 1:**
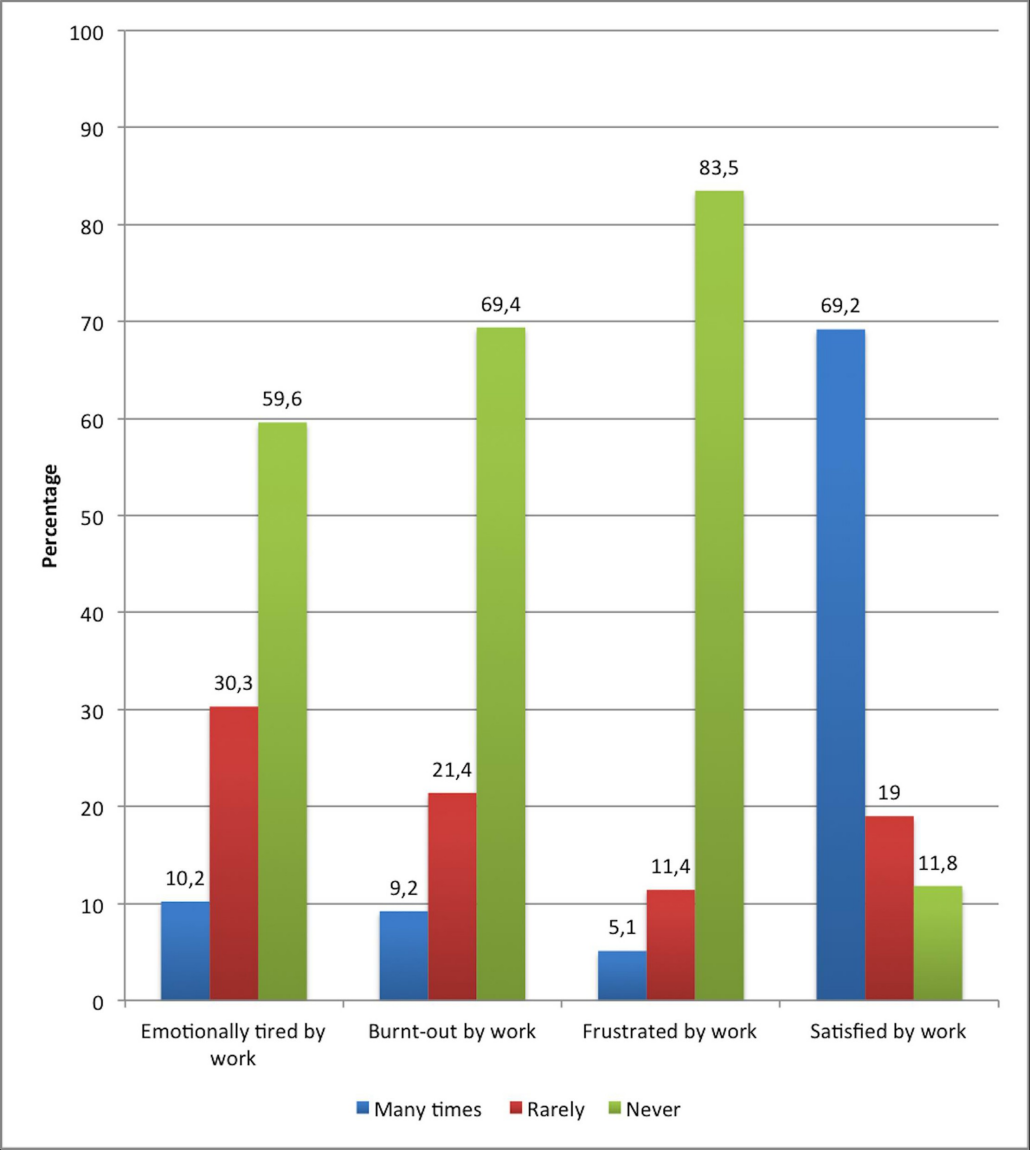
Burnout-related symptomatology among participants, according to their self-rated frequency of appearance.

Overall, it is worth mentioning one problematic fact that emerged from the research: 41.6% of drivers who participated in this study present signs and/or symptoms of emotional exhaustion. Specifically, they claimed to be emotionally tired (10.2%), report burnout symptoms (9.2%), or declare being frustrated with their job (5.1%) (see Fig 1).

There was a set of statistically significant contingences found in this variable for sex (*X*^*2*^ = 7.313; p≤ 0.01) and age (*X*^*2*^ = 14.033; p≤0.05): women (45.9%, *n* = 245) and the group of drivers ranging from 46 to 55 years (46.1%; *n* = 107) showed more signs or symptoms associated with emotional exhaustion (EEW), in contrast to the group of men (38.1%; *n* = 254) and the group over 65 years (23.3%; *n* = 14). The frequency and percentage of drivers with "*Low-mid* and *High* Emotional Exhaustion" were also calculated according to their sex and age (please see Table 3).

**Table 3:** Frequency and percentage of sex and age-based distribution of drivers self-reporting emotional exhaustion at work (EEW).

Level		High		Low-mid	
		Frequency (*n =* 499)	Percentage	Frequency (*n =* 701)	Percentage
**Sex**	Women	245	49.1	289	41.2
	Men	254	50.9	412	58.8
**Age**	18–25	46	9.2	79	11.3
	26–35	116	23.2	149	21.3
	36–45	152	30.5	194	27.7
	46–55	107	21.4	125	17.8
	56–65	64	12.8	108	15.4
	>65	14	2.8	46	6.6

**Fig 2:**
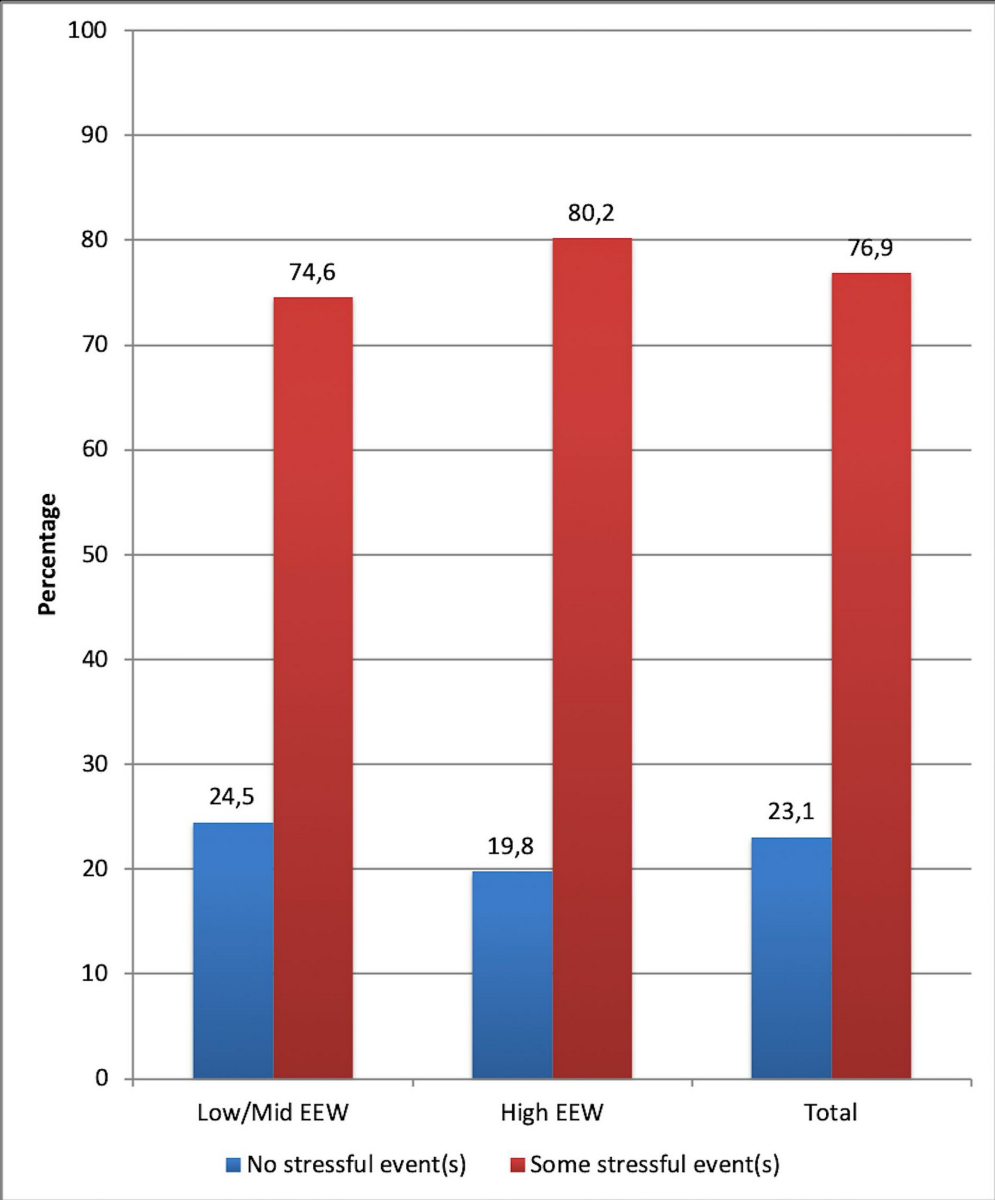
Percentage distribution of the drivers who experienced (or not) stressful events, depending on whether they presented (or not) burnout symptoms, compared to the general distribution of the sample.

Furthermore, 80.2% of drivers reporting symptoms of workplace stress or emotional exhaustion at work had experienced one or more than one stressful life event, among the ones considered in the study, during the previous year (see Fig 2).

Although there are no significant differences or associations, we observed a high percentage of emotional exhaustion in people working in house holding (9.2%) compared to the general average reported by the full sample (7.8%).

Among drivers with fewer EEW levels, it is worth noticing the group composed of individuals with an average value of driving experience higher than 30 years (67.4%; *n* = 145) and those who are currently occupationally inactive (66.2%; *n* = 180).

However, it was not possible to establish significant relationships when crossing the burnout indicator (EEW) with demographic and driving-related variables, such as the size of the city of residence, the objective risk exposure, the vehicle driven, the main reasons for driving, the day/night hours of driving, the periods of non-stop driving, the most used types of road, the crash history, the traffic fines received in the last 3 years (excluding parking fines), the occupation, and the usual work schedule.

The symptoms of emotional exhaustion have proved to be related, with a significant probability, to the fact of not feeling in good conditions to drive (*X*^*2*^ = 5.403, p<0.05).

**Fig 3:**
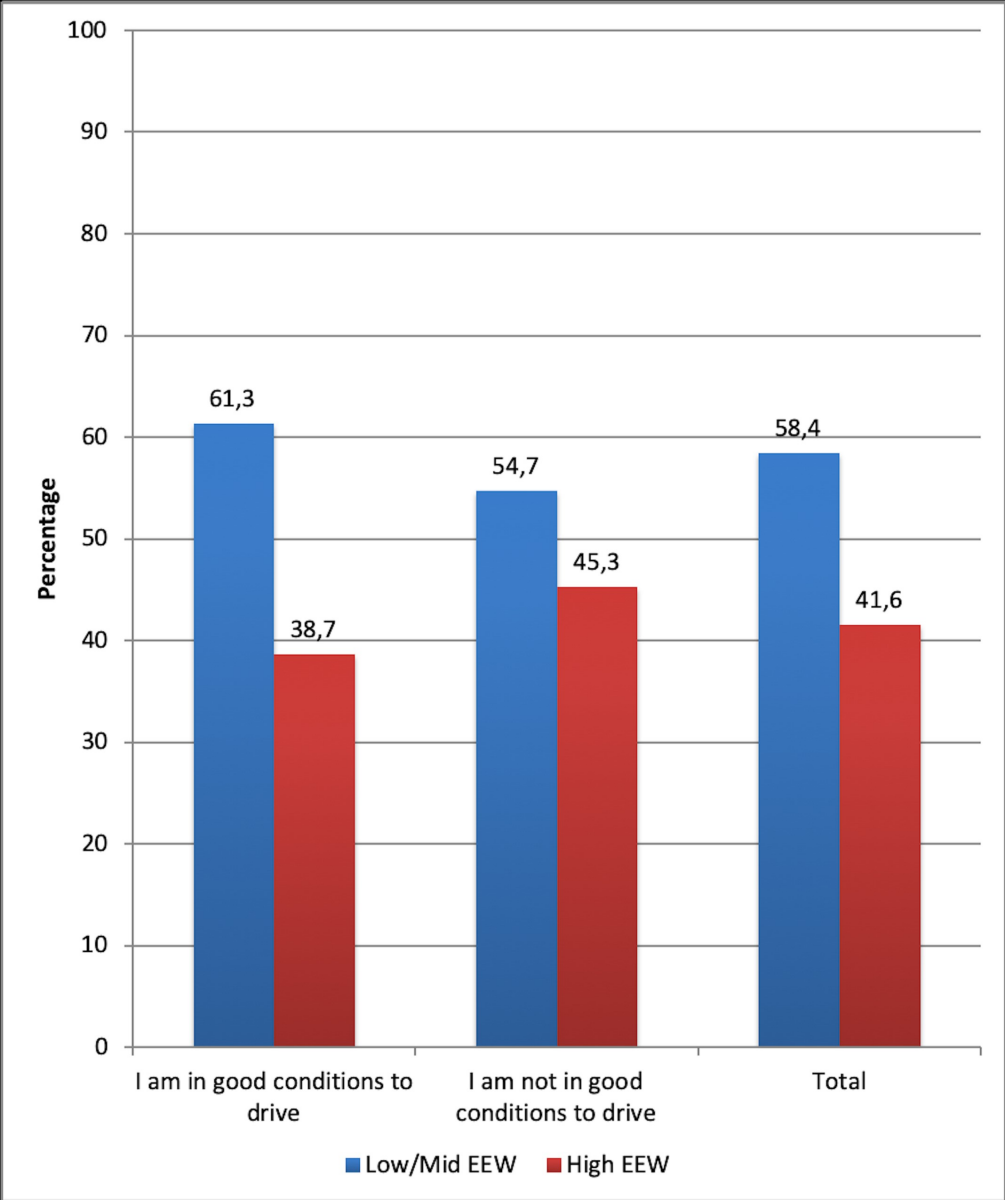
Distribution of participants according to their EEW level, depending on whether they think they are (or not) in good condition to drive.

Specifically, 45.3% (*n* = 238) of the drivers who were not in good condition to drive also showed considerable burnout indices (EEW), in contrast with the other 54.7% (*n* = 287), who did not present symptoms related to EEW, even though they did not feel in good condition to drive (Fig 3).

While 38.7% (*n* = 261) of those who think they were in condition to drive have signs of EEW, this percentage increases to 45.3% (*n* = 238) for those drivers who have often considered that they are not in an appropriate health condition to drive (see Fig 3).

When drivers admitted that they were not in good condition to drive, the level of emotional exhaustion at work was independent from the decision to keep driving, and from the assessment of its effect on driving. Furthermore, significant relationships between EEW and the fact of driving during sick leaves were not found.

We found significant differences in the evaluation of the effects of certain pathologies (e.g. depression, insomnia, need of taking medicines). While all drivers believe that these conditions seriously affect the ability to drive safely, the group of drivers without burnout (EEW) levels regard them as more relevant.

## Discussion

The aim of this study was to assess the relationships among the following elements: two unhealthy work indicators, the decision to drive (or not), and driving crashes suffered by Spanish workers. Overall, the results show how job stress and emotional exhaustion at work are significantly associated with and, in the case of job stress, correlated to the driving crashes they suffered. However, it is also noticeable how job stress and emotional exhaustion, although potentially impairing for the driving performance, do not seem to influence the decision to drive (or not), even when the subject experiences them to a considerable extent.

In regard to the link found between job stress and driving crashes, scientific evidence supports the fact that workplace stress negatively affects the performance of several activities, such as driving [53]. In this sense, it would be convenient to design and apply interventions aimed at training drivers, so that they may be appropriately aware of, identify, accept, and cope with *unhealthy* job-related issues [54]. On the other hand, and unlike what was hypothesized, emotional exhaustion at work (EEW), although associated with job stress (JS), was not significantly linked to the number of driving crashes suffered during the three previous years, even though recent evidence suggests that, for instance, this variable impairs job performance through the statistical mediation of motivational issues [55].

As for the measurement tool used in this study for life-related stressful event (LSE), there was evidence that drivers who have experienced stressful life events during the previous year are more likely to show symptoms related to burnout. The identification of the significant effects of work stressors among drivers is relevant since, despite the fact that the health of drivers at the moment of the survey could be self-reported as "good", repeated exposure to stressful events at work is related to the development of various health problems in the medium and long-term [56,57]. In other words, awareness on both stress (in different spheres) and burnout-related symptoms such as EEW seem to be a relevant factor to consider in interventions, as a manner of protecting drivers from both the prolonged experience and the subsequent negative effects on health, performance and well-being, that these factors may imply [53,54].

Another key issue that should be discussed is the problematic relationship that job stress and EEW seem to keep with driving performance [10,24]. Some studies have documented that the negative effects of stress are caused not only by immediate reactive patterns, but also by the patterns of stress that affect activities such as driving. These patterns could eventually be elicited more easily if the individual was exposed to such stimuli with high frequency or regularity [58]. The occupational context, more specifically, can be described as an ideal scenario for the prevention and training of everything that concerns coping with stress, emotional exhaustion at work and other relevant factors that may influence outcomes in driving safety, such as fatigue and adequate recovery of workers [31,59]. This is due to two facts: first, work is one of the vital scenarios in which stress is most prevalent, and second, it is a potential source of good interventions that may result in lower rates of occupational diseases, work fatigue, and accidents, both inside and outside of the workplace [56,60].

Although the stress generated by working in the field of driving implies a series of potential risks for road safety, occupational stress cases directly related with the constant exposure to stressors on the road may also have serious implications in the prediction of traffic accidents [1,60]. In this regard, recent evidence shows that there are different patterns of involvement in traffic accidents and penalties concerning groups of professional and non-professional drivers, related to stress factors and risky behaviors such as speeding, being aggressive towards other road users, and driving under fatigue-related conditions [60,61]. Indeed, recent researches have documented the actual influence of factors such as fatigue and driver’s demographic features [59] on both risky behaviors performed and severity of traffic crashes suffered [37,42,46,62]. Furthermore, the relative risk of accidents caused by human factors is higher among professional drivers, but other occupations that present various factors related to job strain have highly odd ratios of being involved in road accidents when driving as well [61].

Finally, it is important to note that most Spanish drivers composing the study sample that showed higher levels of EEW often felt, contrary to the expected, in adequate conditions to drive (38.7% of them), even though our results support the significant association between unhealthy work indicators (in this case, job stress) and risky driving behaviors. In this regard, a possible measure aimed at strengthening the road safety of drivers from the occupational sphere could be to train workers in key issues potentially impairing their driving performance, such as the recognition of stress and emotional exhaustion -and their influence in the crash risk-, emotional coping and decision-making under stress-related symptoms.

## Conclusions

The results of this study show that, overall, a high percentage of the Spanish working population presents considerable indices of job stress and emotional exhaustion at work.

Furthermore, the significant relationships found between levels of emotional exhaustion, job stress and traffic crashes suffered by drivers support the hypothesis that unhealthy work-related factors have an impact on driving performance.

However, the decision to drive (or not) does not seem to be significantly affected by the scores obtained in JS and EEW, thus raising the question about whether there is, indeed, no relationship between these variables; or, rather, whether there is no full awareness among workers about job stress and emotional exhaustion symptoms, and their relationship with the driving performance.

All these data suggest that there is an urgent need of interventions in this field, in order to enhance occupational strategies aimed at the management of psychosocial work factors, including job stress and emotional exhaustion at work. Also, and considering that traffic crashes suffered by workers commonly take place outside the workplace, evidence-based interventions in this field may contribute to strengthen road safety and public health settings.

## Limitations of the study and further research

One of the most important limitations of this work is related to the use of self-reports as primary source of information, fact that could potentially lead to several biases such as social desirability [63], or simply to an inefficient understanding of the questions, especially among older drivers. In this regard, and as a recommendation for future research experiences, we should remark that it is important to use both objective and subjective measures for the assessment of driving perceptions and for stress/burnout supplementary indicators (including the positive ones, such as the personal accomplishment at work), since they help minimize the so called *common method biases* typically affecting cross-sectional research designs [64].

Furthermore, there are some work environment-related variables which were not evaluated in this study, such as the psychological and physical demands of the job and the perceived social support from colleagues and supervisors that, in the light of (for instance) approaches such as the Job Demand-Control model [19], could be useful to deepen the discussion about traffic outcomes and their relation with the workplace characteristics of drivers.

Finally, future research could include perceptions and complementary observations on how drivers cope with job stress according to demographical variables (e.g. age, sex, social and physical appearance), considering the target groups identified in this study.

## Supporting information

S1 Dataset Raw data are available in the file (database) attached to the electronic version of this manuscript. (ZIP)
